# Capsular Typing Method for Streptococcus agalactiae Using Whole-Genome Sequence Data

**DOI:** 10.1128/JCM.03142-15

**Published:** 2016-04-25

**Authors:** Anna E. Sheppard, Alison Vaughan, Nicola Jones, Paul Turner, Claudia Turner, Androulla Efstratiou, Darshana Patel, Modernising Medical Microbiology Informatics Group, A. Sarah Walker, James A. Berkley, Derrick W. Crook, Anna C. Seale

**Affiliations:** a Modernising Medical Microbiology Consortium, Nuffield Department of Clinical Medicine, University of Oxford, Oxford, United Kingdom; b Centre for Tropical Medicine, Nuffield Department of Clinical Medicine, University of Oxford, Oxford, United Kingdom; c Shoklo Malaria Research Unit, Mae Sot, Thailand; d Microbiology Reference Division, Public Health England, London, United Kingdom; e Imperial College, London, United Kingdom; f KEMRI-Wellcome Trust Research Programme, Kilifi, Kenya

## Abstract

Group B streptococcus (GBS) capsular serotypes are major determinants of virulence and affect potential vaccine coverage. Here we report a whole-genome-sequencing-based method for GBS serotype assignment. This method shows strong agreement (kappa of 0.92) with conventional methods and increased serotype assignment (100%) to all 10 capsular types.

## TEXT

Streptococcus agalactiae, or group B streptococcus (GBS), is an important pathogen in neonates (1–3), with early infections being acquired from the maternal genitourinary tract (4). In addition, GBS is now recognized as an increasingly important pathogen among immunosuppressed and elderly individuals in high-income regions (5, 6).

GBS expresses a capsular polysaccharide that is involved in virulence and immune evasion. Ten different serotype variants (i.e., Ia, Ib, II, III, IV, V VI, VII, VIII, and IX), which differ in their disease-causing abilities, have been described. Conjugate vaccines targeting the most common disease-causing serotypes are currently in development (7). Establishment of vaccine serotype coverage is important, as is postintroduction surveillance to monitor for potential serotype replacement, as has been seen following the introduction of other conjugate vaccines (8).

Current methods for GBS serotype allocation rely on latex agglutination assays or PCR assays (9). Recent advances in whole-genome sequencing (WGS) have enabled the development of approaches that can be used in place of traditional microbiological methods, such as strain typing and antibiotic susceptibility profiling (10–12). A major advantage of this approach is that the cost of sequencing can be mitigated by the ability to use the same data to generate multiple outputs. Given the decreasing cost of WGS (13) and the ongoing increase in WGS data generation, we sought to establish and to validate a WGS-based method for GBS capsular typing.

We developed an algorithm for serotype assignment on the basis of sequence similarity between a given *de novo* assembly and capsular gene sequences of the 10 GBS serotypes. For nine serotypes, published sequences were used as references (Table 1); for serotype IX, however, only a partial capsular locus sequence has been published (14). A suitable reference for the full capsular locus region was therefore determined by WGS of a serotype IX isolate obtained from the Statens Serum Institute (Copenhagen, Denmark).

**TABLE 1:** Reference sequences used for sequence-based serotype allocation

Serotype	GenBank accession no.	Region (bp)	Reference
Ia	AB028896.2	6982–11695	Yamamoto et al. (20)
Ib	AB050723.1	2264–6880	Watanabe et al. (21)
II	EF990365.1	1915–8221	Martins et al. (22)
III	AF163833.1	6592–11193	Chaffin et al. (23)
IV	AF355776.1	6417–11656	Cieslewicz et al. (24)
V	AF349539.1	6400–12547	Cieslewicz et al. (24)
VI	AF337958.1	6437–10913	Cieslewicz et al. (24)
VII	AY376403.1	3403–8666	Cieslewicz et al. (24)
VIII	AY375363.1	2971–7340	Cieslewicz et al. (24)
IX	NA^*a*^	NA	This study

a NA, not applicable.

To assign the serotype for a given isolate, a BLAST database was generated from the *de novo* assembly and queried with the variable region of the capsular locus sequence for each serotype (*cpsG-cpsK* for serotypes Ia to VII and IX and *cpsR-cpsK* for serotype VIII), using BLASTn with an E value threshold of 1e−100 and otherwise default parameters. A serotype was considered correct if it showed ≥95% sequence identity over ≥90% of the sequence length. These thresholds were chosen on the basis of being stringent enough to provide differentiation between the various reference sequences while maximizing serotype allocation for an initial test set of publicly available GBS WGS data, for which serotype information was not available (therefore, we had no way of knowing whether the assigned serotypes were actually correct).

This sequence-based method for serotype allocation was validated using WGS with a set of 223 colonizing or invasive human isolates from Canada, Latin America, Singapore, the United Kingdom, the United States, and Thailand for which serotypes had been determined previously using conventional latex agglutination assays, with PCR assays being used to confirm weak positive or negative results in a subset (15–17). For two rare serotypes (serotypes VIII and IX), one isolate of each was obtained from the Statens Serum Institute. GBS isolates stored at −80°C were subcultured on Columbia blood agar for 24 to 48 h, followed by DNA extraction from a single colony using a commercial kit (QuickGene; Fujifilm, Tokyo, Japan). High-throughput sequencing was performed at the Wellcome Trust Centre for Human Genetics (Oxford University, Oxford, United Kingdom) using the Illumina HiSeq2500 platform, generating 150-base paired-end reads. *De novo* assembly was performed using Velvet and VelvetOptimiser (18, 19). Agreement between serotype allocations was tested with the kappa statistic.

**FIG 1:**
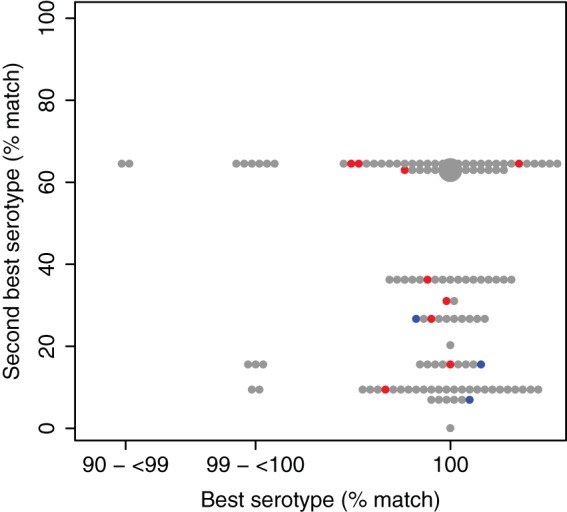
Relationships between allocated serotypes (best matches) and second-best matches. For each isolate, the percentage of the capsular locus region present (≥95% sequence identity) for the assigned serotype is shown on the *x* axis and that for the serotype showing the next best match is shown on the *y* axis. Gray circles, isolates showing agreement between sequence-based serotyping and conventional serotyping; blue circles, isolates classified as nontypeable by conventional methods; red circles, isolates with discordant results. Small circles, single isolates; large circle, 100 isolates. For each serotype, the second-best match is identical in all cases, leading to the observed horizontal banding (details in Table 3).

High-quality sequence data were obtained for all 223 GBS isolates (median read number, 2,975,508 [range, 1,798,744 to 13,073,718]; median contig number, 46 [range, 16 to 106]; median assembly length, 2.05 Mb [range, 1.94 to 2.22 Mb]). Each isolate was allocated to a single serotype using the WGS data (Table 2). Three isolates that did not have a capsular type assigned by latex agglutination methods had serotypes Ib, VI, and VII assigned. For all previously serotyped GBS isolates with a known capsule type, the kappa statistic of 0.92 indicated very strong agreement between WGS-predicted and conventional serotypes. Nine isolates had discordant results. In each case, there was strong support for the sequence-allocated serotype, with >98% sequence identity over 100% of the reference length in all nine cases (Fig. 1). Across all isolates, differences in relatedness between the capsular locus sequences of the different serotypes led to characteristic relationships between the allocated serotype (best match) and the second-best match. For example, all isolates assigned to serotype Ia had serotype III as the second-best match. In all cases, the second-best match was substantially poorer than the best match, demonstrating that there was no ambiguity in the predicted serotype (Fig. 1 and Table 3).

**TABLE 2:** Serotype allocation by WGS versus serotype allocation by latex agglutination

Latex agglutination serotype	No. with WGS serotype of:										Total no.
	Ia	Ib	II	III	IV	V	VI	VII	VIII	IX	
Ia	34	0	0	1	0	0	0	0	0	0	35
Ib	0	9	1	0	0	0	0	0	0	0	10
II	0	0	25	0	0	0	0	0	0	0	25
III	3	0	0	111	0	0	0	0	0	1	115
IV	0	0	0	0	1	0	1	0	0	0	2
V	0	0	0	0	0	16	0	0	0	0	16
VI	0	0	0	0	0	1	8	0	0	0	9
VII	0	0	0	0	0	0	0	5	0	0	5
VIII	0	0	0	0	0	0	0	0	1^*a*^	0	1
IX	0	1	0	0	0	0	0	0	0	1^*a*^	2
Nontypeable	0	1	0	0	0	0	1	1	0	0	3
Total	37	11	26	112	1	17	10	6	1	2	223

a Reference GBS isolates from Statens Serum Institute for serotypes VIII and IX.

**TABLE 3:** Relationships between allocated serotypes and second-best matches^*a*^

Allocated serotype	Match (%) for allocated serotype	Second-best serotype	Match (%) for second-best serotype
Ia	93.91–100	III	64.56
III	100	Ia	62.98
V	100	IX	36.26
IX	100	V	31.05
VI	100	III	26.68
IV	100	Ia	20.3
Ib	99.61–100	VI	15.55
II	99.86–100	IV	9.45
VII	100	Ib	6.95
VIII	100	None	0

a See also Fig. 1.

The nine isolates with discordant results and the three nontypeable isolates were retested by latex agglutination assays (Table 4) and were resequenced using the Illumina MiSeq platform, with sequence processing and WGS-based serotype prediction performed as described above. In all cases, resequencing results were consistent with the initial WGS classification. For 6/9 isolates with discordant results, the new latex agglutination results matched the WGS-based prediction, suggesting that the initial discordance might have resulted from incorrect latex agglutination typing or sample mislabeling. The other three isolates with discordant results and the three nontypeable isolates were all classified as nontypeable with retesting.

**TABLE 4:** Retyping of isolates with discordant results and nontypeable isolates

Isolate	Reason for retyping	Latex agglutination serotype		WGS serotype	
		Initial	Repeat	Initial	Repeat
CB466	Discordant results	III	Ia	Ia	Ia
IW8194	Discordant results	III	IX	IX	IX
IW8466	Discordant results	Ia	III	III	III
IW8471	Discordant results	III	Ia	Ia	Ia
IW7157	Discordant results	Ib	II	II	II
SMRU1	Discordant results	VI	V	V	V
SMRU25	Discordant results	IV	NT^*a*^	VI	VI
SMRU4	Discordant results	IX	NT	Ib	Ib
SMRU59	Discordant results	III	NT	Ia	Ia
Z41	NT	NT	NT	Ib	Ib
UK22	NT	NT	NT	VII	VII
IW2723	NT	NT	NT	VI	VI
CB454	Control	III	III	III	III
IW4445	Control	Ia	Ia	Ia	Ia
IW4077	Control	II	II	II	II

a NT, nontypeable.

This WGS-based method for GBS serotyping, which was validated using 223 isolates that had been typed using conventional methods, was therefore highly accurate. Although WGS currently may not be cost-effective for direct replacement of traditional serotyping, costs are likely to decrease further. Furthermore, WGS may already be the cheapest option for combined studies, with possibilities for utilizing the resulting data for additional analyses, such as multilocus sequence typing, analyses of relatedness to other sequenced isolates, and detailed phylogenetic analyses.
